# Development and challenges of palliative care in Indonesia: role of psychosomatic medicine

**DOI:** 10.1186/s13030-017-0114-8

**Published:** 2017-11-21

**Authors:** Rudi Putranto, Endjad Mudjaddid, Hamzah Shatri, Mizanul Adli, Diah Martina

**Affiliations:** Division of Psychosomatic and Palliative Medicine, Department of Internal Medicine, Dr. Cipto Mangunkusumo National General Hospital – Universitas Indonesia, Jl. Diponegoro No. 71, Jakarta Pusat, 10430 Indonesia

**Keywords:** Healthcare, Palliative care, Psychosomatic medicine

## Abstract

**Purpose of review:**

To summarize the current status of palliative care and the role of psychosomatic medicine in Indonesia.

**Recent findings:**

Palliative care is not a new issue in Indonesia, which has been improving palliative care since 1992 and developed a palliative care policy in 2007 that was launched by the Indonesian Ministry of Health. However, the progress has been slow and varied across the country. Currently, palliative care services are only available in a few major cities, where most of the facilities for cancer treatment are located. Psychosomatic medical doctors have advantages that contribute to palliative care because of their special training in communication skills to deal with patients from the standpoints of both mind and body.

**Summary:**

Palliative care services in Indonesia are established in some hospitals. Future work is needed to build capacity, advocate to stakeholders, create care models that provide services in the community, and to increase the palliative care workforce. Psychosomatic medicine plays an important role in palliative care services.

## Background

The Ministry of Health of Indonesia has predicted around 240.000 new cases of cancer per year, with 70% of the patients already incurable at the time of diagnosis. There are a small number of excellent facilities, especially for cancer, however the rest of the facilities operate with limited resources. Palliative care has not yet developed in most areas. Until recently, most cancer patients eventually died in hospital, suffering unnecessarily due to a high burden of symptoms and unmet needs of the patients and their families [1] Grassi et al. reported that in palliative care and oncology settings there are several dimensions, including emotional distress, anxiety and depression, maladaptive coping, and dysfunctional attachment, that need a broad psychosomatic approach [2].

A study in Japan that described the role of psychosomatic medicine doctors in palliative care has been published [3]. Herein, the authors describe the role of psychosomatic medicine in developing palliative care in Indonesia.

## Overview of palliative care

The World Health Organization (WHO) defined palliative care as an approach that improves the quality of life of patients (adults and children) and their families who are facing problems associated with life-threatening illness. It prevents and relieves suffering through the early identification, correct assessment, and treatment of pain and other problems. Palliative care is the prevention and relief of suffering of any kind – physical, psychological, social, or spiritual – experienced by adults and children living with life-limiting health problems. It promotes dignity, quality of life and adjustment to progressive illnesses, using the best available evidence [4].

In 2014, a 67th World Health Assembly resolution, WHA 67.19, on palliative care recognized that the limited availability of palliative care services in much of the world leads to great, avoidable suffering for millions of patients and their families [4].

Addressing suffering involves taking care of issues beyond physical symptoms. Palliative care uses a team approach to support patients and their caregivers. This includes addressing practical needs and providing bereavement consulting. It offers a support system to help patients live as actively as possible until death. Palliative care is explicitly recognized under the human right to health. It should be provided through person-centered and integrated health services that pay special attention to the specific needs and preferences of individuals. Palliative care is required for a wide range of diseases. The WHO reported that the majority of adults in need of palliative care have chronic diseases such as cardiovascular diseases, cancer, chronic respiratory diseases, AIDS, and diabetes (4.6%). Many other conditions may require palliative care, including kidney failure, chronic liver disease, multiple sclerosis, Parkinson’s disease, rheumatoid arthritis, neurological disease, dementia, congenital anomalies, and drug-resistant tuberculosis [5].

A study in 2011 of 234 countries, territories, and areas found that palliative care services were only well integrated in 20 countries, while 42% had no palliative care services at all and a further 32% had only isolated palliative care services [6].

Palliative care is most effective when considered early in the course of the illness. Early palliative care not only improves quality of life for patients but also reduces unnecessary hospitalizations and use of health-care services [4].

## Overview palliative care in Indonesia

Actually, palliative care is not a new issue in Indonesia, where we have been developing palliative care since 1992. However, the progress has been very slow and varied across the country. Currently, palliative care services are available only in several major cities where most of the facilities for cancer are located. The progress of these centers varies in terms of personnel, facilities, and the types of service delivery. We should say that the palliative care concept and implementation are not really understood by some health care practitioners; however, the basic concept is understood by many.

A worldwide report in 2015 by The Economist Intelligence Unit (EIU) about the Quality of Death Index ranked Indonesia 53rd in palliative care (Table 1). This measure was done of 80 countries around the world. Its ranking is based on comprehensive national policies, the extensive integration of palliative care into the National Health Service, a strong hospice movement, and deep community engagement on the issue [7].

**Table 1:** Ranking palliative care across the World

Rank	Country	Value
1	UK	93.9
2	Australia	91.6
3	New Zealand	87.6
4	Ireland	85.8
5	Belgium	84.5
6	Taiwan	83.1
7	Germany	82
8	Netherlands	80.9
9	US	80.8
10	France	79.4
11	Canada	77.8
12	Singapore	77.6
53	Indonesia	33.6
54	Tanzania	33.4
67	India	26.8
80	Iraq	12.5

Adapted from The Economist Intelligence Unit, 2015 [7]

### Palliative care providers

WHO (2016) reported that palliative care is best delivered through a multidisciplinary team. Providers at all levels of care, from palliative care medical specialists to trained volunteers, work together to ensure the best quality of life for the patient. In tertiary care settings, most of them include oncologists, internal medicine physicians, radiotherapists, radiotherapy technicians, psychologists or counselors, nutritionists, physiotherapists, oncology nurses, pharmacists, social workers, and palliative care nurses.

In resource-poor settings, community health workers or trained volunteers – supported, trained and supervised by primary- and secondary-level health-care professionals – are the principal providers of palliative care. Trained community nurses (auxiliary nurses/palliative nursing aides), if permissible within the system, can play a major role in delivery of care. Family members also have a large role in caring for patients at home, and they need to be supported.

Primary care and community care are essential to provide palliative care services to the large majority of people in need. Much of the care of dying persons has to occur in the community and in all health-care settings, mainly conducted by health professionals who are generalists and not specialist practitioners. Most people with advanced chronic conditions with palliative care needs are living in the community, so primary care professionals should be able to identify and care them [3]. A study by Kristanti MS et al. reported that family caregivers can enhance the quality of life for palliative care cancer after getting basic skill training in palliative care [8].

Currently, Cipto Mangunkusumo Hospital and other district hospitals in Jakarta are participating with Singapore International Foundation (SIF) and Singapore International Volunteers in partnership with the Jakarta Cancer Foundation and Rachel House to organize a three-year training program for doctors, nurses, and pharmacists working at public hospitals in Jakarta with the aim of improving care for the terminally ill in Jakarta province. SIF’s “Enhancing palliative Care Practice” project aims to make possible the sharing of palliative care knowledge and skill between the medical communities of two countries through capacity building activities and professional sharing platform. However the participants are still limited and it is not widely available to all clinicians in Indonesia.

### Pain management practices

Pain is one of the most frequent and serious symptoms experienced by patients in need of palliative care. Opioid analgesics are essential for treating the pain associated with many advanced progressive conditions. For example, 80% of patients with AIDS or cancer and 67% of patients with cardiovascular disease or chronic obstructive pulmonary disease will experience moderate to severe pain at the end of their lives. Opioids can also alleviate other common distressing physical symptoms including shortness of breath. Controlling such symptoms at an early stage is an ethical duty to relieve suffering and to respect the dignity of ill people [3].

International Narcotics Control Board reported that the levels of consumption of opioids for pain relief in over 121 countries were “inadequate” or “very inadequate” to meet basic medical needs. A study in 2011 found that 83% of the world’s population live in countries with low to non-existent access to opioid pain relief [9]. Setiabudy R et al. reported that Indonesia has an annual consumption rate of only 0.054 mg/capita for pain relief, making it among the worst ranking countries in the world. This indicates that opioids are extremely underused for their correct indications in Indonesia [10].

We use national guidance in pain management based on WHO stepladder analgesia. In 2016, the Ministry of Health issued a national standard for cancer palliative management and, as stated previously, morphine is only available in the hospital setting. There are several preparations available, such as immediate release and sustained release morphine (tablets), fentanyl patch, and intravenous morphine or fentanyl. Every doctor who has a medical practice license is permitted to write prescriptions for morphine.

However, the presence of pain affects all aspects of an individual’s functioning. As a consequence, an interdisciplinary approach that incorporates the knowledge and skills of a number of health care providers is essential for successful treatment and patient management. Treating problematic pain with drugs is not sufficient. The psychosomatic approach has been shown to be useful in treating patients with pain.

### Challenges in developing palliative care in Indonesia

Witjaksono M et al. (2014) reported that palliative care in Indonesia was first established in 1992 in Surabaya, East Java Province. However, its development has been very slow.

Currently, palliative care services are only available in big cities, where most facilities for cancer treatment are located. Among these centers, palliative care is in different stages of development in terms of human resources, facilities, and types of service delivery. The challenges in developing palliative care in Indonesia can be related to government policy, lack of palliative care education, attitudes of health care professionals, and general social conditions in the country. Rochmawati E et al. has reviewed the facilitating factors supporting the provision of palliative care in Indonesia, which include: a culture of strong familial support, government policy support, and volunteering and support from regional organizations [11]. Palliative care has been integrated in the National Cancer Control Program in the period from 2014 to 2019.

In 2007, the Minister of Health announced a national policy on palliative care. However, the policy has not been fully implemented in the health care system due to the absence of palliative care guidelines and standards, a proper referral system, and sufficient funding. Local government plays an important role in developing palliative care. In Surabaya, the Pusat Pengembangan Paliatif dan Bebas Nyeri (PPPBN), a center for palliative care and pain relief based at Dr. Soetomo hospital, was established in 1992. In Jakarta, 12 hospitals have had basic training by Singapore International Foundation in cooperation with Cancer Foundation Jakarta since 2015. However, due to a shortage of physicians certified in palliative care, these are not well developed. Government policy on opioids has also become a significant barrier to providing palliative care [11].

Barriers to the development of palliative care also come from health care professionals. Palliative care is regarded as an option only when active treatment is no longer continued. Psychological problems, social difficulties, and spiritual aspects are not considered to be part of medical services at the end of life. Fighting to the end, not discussing death and dying, opioid phobia, and possible loss of control and income have resulted in a resistance to the referral of patients to palliative care.

Finally, barriers come from the general condition of the country and society such as the wide geographic area and the heterogeneous society, low public awareness of palliative care, taboos around death and disclosure of prognosis, family decision making, reliance on traditional medicines, opioid phobia, and a desire for a curative treatment at all cost.

The development of palliative care in Indonesia has been supported by regional organizations of palliative care such as the Asia Pacific Hospice and Palliative Care Network (APHN) and the Lien Centre for Palliative Care, Singapore. The First International Conference in Palliative Care was successfully held during the 5th Indonesian Palliative Society Congress and in conjunction with the 12th APHN Council Meeting in Yogyakarta from 20 to 22 September 2012. More than 250 doctors and nurses and 150 volunteers attended. APHN country and sector members such as Singapore, Malaysia, India, Australia, Hong Kong, and Taiwan supported this event [12]. On March 2017, the Indonesia Society of Psychosomatic Medicine (ISPM) in collaboration with the Indonesian Society of Hematology Medical Oncology, the Indonesia Palliative Society – Jakarta, and the Indonesia Society of Oncology and supported by the American Society of Clinical Oncology (ASCO) was successful in presenting the 1st International Palliative Care Workshop in Jakarta, Indonesia. More than 80 participants, such as doctors and nurses, attended this event. The meetings brought a positive impact to the development of palliative care in Indonesia.

### Role of psychosomatic medicine in palliative care

**Fig. 1:**
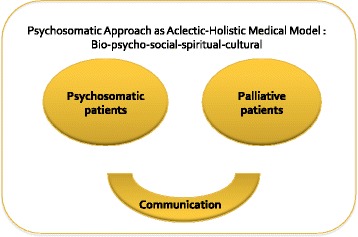
Holistic medical model for psychosomatic and palliative care. A bio-psychosocial-spiritual model for psychosomatic and palliative care patients that is based on bio-physical and psychosocial factors and on good communication [3]

The psychosomatic medicine approach is an interdisciplinary, holistic study of physical and mental disease with a biological, psychosocial, and social-cultural base. Within the interdisciplinary framework of psychosomatic medicine for the assessment of psychosocial factors affecting individual vulnerability, holistic consideration in clinical practice and integration of psychological therapies in the prevention, treatment, and rehabilitation of medical disease are important components [13–15]. Matsuoka H, et al. reported that psychosomatic medicine is based on a bio-psychosocial-spiritual model of care and that it is related to physical and psychosocial factors and good communication. There are many similar aspects between psychosomatic and palliative patients [3], as shown in Fig. 1.

The type of doctor-patient communication in Indonesia is paternalistic because the majority of patients perceive their doctors to be of higher status and thus use a non-assertive style of communication during consultations to show respect and avoid conflict. Patients and their families tend to be afraid of asking too many questions, even though there are things they want to know further.

Regarding decision-making and delivering bad news associated with diagnosis or prognosis, collusion has been accepted as a common practice among doctors and their patient’s family. In Eastern countries, as well as Indonesia, disease is considered a “family matter”, so decision-making is centered on family discussion/joint decision. The belief that knowing about a terminal diagnosis and a poor prognosis would extinguish the patient’s hope and increase anxiety and depression causes the family to “protect” their beloved one from the truth of the illness.

The distress of palliative care patients is a total suffering that is a complicated combination of physical, psychological, social, and spiritual pain, thus whole person care is needed. Psychosomatic medical doctors have the advantage of contributing to palliative care without a stress overload or burnout because of their special training in communication skills to deal with patients from the standpoints of both mind and body.

In the Faculty of Medicine at Universitas Indonesia/Dr. Cipto Mangunkusumo National General Hospital, internal medicine and psychosomatic programs have been teaching palliative and terminal care during their education and services since 2000. Consultants in psychosomatic medicine have been distributed to a few citiies, such as Medan in North Sumatera, Padang in West Sumatera, Palembang in South Sumatera, and Jogjakarta in Central Java. In the future, new psychosomatic clinics will be built in Banda Aceh, Makassar, and Solo. The Indonesian Society of Psychosomatic Medicine was actively involved in developing palliative care in Indonesia.

The assessment of other psychosocial dimensions among cancer and palliative patients has been raised by research in oncology [16]. Fava et al. reported that The Diagnostic Criteria for Psychosomatic Research consists of twelve clinical clusters that explore a variety of possible psychological conditions and emotional responses to medical illness [17].

A study by Mahendran R et al. that focuses on hope in the Asian cancer population showed that biopsychosocial factors that were most consistently associated with hope and hopelessness included socio-demographic variables (education, employment and economic status); clinical factors (cancer stage, physical condition and symptoms); and psychosocial factors (emotional distress, social support and connections, quality of life, control or self-efficacy, as well as adjustment and resilience) [18].

Psychosomatic and internal medicine doctors should be role models and work together with other specialty and health workers in the development of palliative care in Indonesia.

## Conclusions

Palliative care is not a new issue in Indonesia, which has been improving palliative care since 1992 and developed a palliative care policy in 2007 that was launched by the Indonesian Ministry of Health. However, progress has been slow and varied across the country. Future work is needed to build capacity, advocate to stakeholders, and to create care models that provide services in the community and increase the palliative care workforce. Psychosomatic medicine is based on a bio-psychosocial-spiritual model of care and is related to physical and psychosocial factors and to good communication. There are many similarities between psychosomatic and palliative patients. Psychosomatic medical doctors have the advantage of contributing to palliative care without stress overload or burnout because of their special training in communication skills to deal with patients from the standpoints of both mind and body.

## Abbreviations

AIDS: Acquired Immune Deficiency Syndrome
APHN: Asia Pacific Hospice Network
ASCO: American Society of Clinical Oncology
EIU: Economist Intelligent Unit
ISPM: Indonesian Society of Psychosomatic Medicine
MPI: Masyarakat Paliatif Indonesia
PPPBN: Pusat Pengembangan Paliatif dan Bebas Nyeri
SIF: Singapore International Foundation
WHA: World Health Assembly
WHO: World Heath Organization
